# Compliance with the Dietary Approaches to Stop Hypertension (DASH) Diet: A Systematic Review

**DOI:** 10.1371/journal.pone.0078412

**Published:** 2013-10-30

**Authors:** Mandy Wing-Man Kwan, Martin Chi-Sang Wong, Harry Hao-Xiang Wang, Kirin Qi-Lin Liu, Catherine Lok-Sze Lee, Bryan Ping-Yen Yan, Cheuk-Man Yu, Sian Meryl Griffiths

**Affiliations:** 1 School of Public Health and Primary Care, Faculty of Medicine, The Chinese University of Hong Kong, Hong Kong SAR, China; 2 General Practice and Primary Care, Institute of Health and Wellbeing, University of Glasgow, Glasgow, United Kingdom; 3 Department of Medicine and Therapeutics, Faculty of Medicine, The Chinese University of Hong Kong, Hong Kong SAR China; Universidad Peruana de Ciencias Aplicadas (UPC), Peru

## Abstract

**Background:**

The Dietary Approaches to Stop Hypertension (DASH) diet has been recognized as effective to lower blood pressure in feeding trials, but compliance with the diet must be persistent to maximize health benefits in clinical practice. This paper reports a systematic review of the latest evidence on the method to assess DASH compliance and the corresponding patients' compliance in interventional settings.

**Methods:**

The databases including MEDLINE, EBM Reviews, EMBASE, and CINAHL Plus were searched for original research studies published in the period of January 1992- December 2012 that evaluated compliance with DASH diet. Studies written in English language, with DASH intervention, with complete documentation of the degree of DASH compliance and the assessment method used were included in this review. The search terms included: dietary approaches to stop hypertension, DASH, compliance, adherence, consistency, and concordance.

**Results:**

Nine studies were included. Different types of interventions were identified, ranging from feeding trial to dietary counseling. These studies differed in the assessment methods used to evaluate DASH compliance, which included objective approaches like measurement of urinary excretion, and subjective approaches like dietary intake assessment for DASH target comparison and construction of DASH scoring systems. Compliance levels were lower in educational interventions than that of the original DASH feeding trial.

**Conclusions:**

To conclude, although no consensus existed regarding the best approach to assess DASH compliance, its suboptimal compliance warrants attention. This study implied a need to investigate effective approaches to sustain the DASH dietary pattern beyond counselling alone.

## Introduction

According to the WHO Health Statistics 2012, the global prevalence of raised blood pressure (BP) (systolic blood pressure (SBP): ≥140, diastolic blood pressure (DBP): ≥90) in males and females aged ≥25 years was 29.2% and 24.8%, respectively [1]. Similar percentages were observed in the Hong Kong Population Health Survey 2003/2004, in which the overall hypertension prevalence among people aged ≥15 was 27.2%, with 30.2% in males and 24.9% in females [2].

Globally, increased BP has been reported as the top behavioral and physiological risk factor (13%) for attributable deaths [1]. By estimation, 51% of stroke deaths and 45% of coronary heart disease deaths were caused by increased BP [1]. Besides, high BP also increases the chance of having heart attack, heart failure, stroke, and kidney disease. The positive association between BP and cardiovascular diseases has been supported by consistent evidence [3]. Prospective observational studies estimated a 5–6 mmHg increase in DBP could lead to increased risk of stroke and ischemic heart disease by approximately 35–40% and 20–25%, respectively [4]. Given such global burden of disease, it is of high importance to achieve optimal BP control in the population.

The Dietary Approaches to Stop Hypertension (DASH) is currently recommended as one of the essential lifestyle measures for controlling BP in international guidelines [3], [5]–[7]. It is a diet which recommended higher consumption of whole grain, fruits and vegetables, low-fat dairy products, lean meat, poultry and fish, and nuts and legumes. It is rich in potassium, magnesium, calcium, and dietary fiber, while limiting the intake of total fat, saturated fat and cholesterol. Daily dietary sodium is restricted to 2300 mg. The original DASH feeding trial, in which subjects were provided with all their food, demonstrated a significant reduction of SBP and DBP in patients with stage 1 hypertension by 11.4 and 5.5 mmHg net of the control, respectively (*p*<0.001 for both) [8]. Such BP lowering effect occurred within 2 weeks. Other intervention studies focusing on dietary education, which included face-to-face dietary counseling by dietitian, phone calls, and mailings, also observed a significant reduction of SBP and DBP ranged 5.6–11.2 mmHg and 4.1–7.5 mmHg respectively [9]–[11].

However, although the efficacy of DASH diet to effectively lower BP has been demonstrated, information regarding the assessment methods and degree of compliance to DASH diet following dietary intervention is limited. The major landmark studies on DASH diet are feeding trials with all food provided to the participants, which may limit generalization of the findings to clinical settings where intervention is mainly based on counseling. A better understanding on the methods used to examine compliance will help researchers to evaluate the impact of the intervention, and hence to develop effective strategies to improve the dietary compliance and optimize patient outcomes. The aim of this study was to systematically review the assessment methods used in previous DASH trials and the corresponding compliance level to DASH diet among the participants.

## Methods

### Search strategies

The study was approved by the Clinical Research Ethics Committee, The Chinese University of Hong Kong. Systematic literature searches were conducted in databases including MEDLINE, EBM Reviews (Cochrane Central Register of Controlled Trials, Cochrane Database of Systematic Reviews, and Cochrane methodology Register), EMBASE, and CINAHL Plus for eligible studies that reported compliance to the DASH diet between Jan 1992- Dec 2012. Since the first DASH study was conducted in 1993, using the search cut-off date as of 1992 could ensure all the relevant studies could be identified in this search. Two independent investigators developed the search strategies. Search phrase (DASH OR dietary approaches to stop hypertension) AND (compliance OR adherence OR consistency OR concordance) was used to search all of the fields in the above database (see **Table S1**). An additional manual search was performed using reference lists from the research studies and review articles to identify other potential eligible studies.

### Selection of studies and quality assessment

Studies that fulfilled the following criteria were eligible for inclusion: (1) an randomized controlled trial design; (2) conducted in humans; (3) written in English; (4) with a study arm of DASH intervention; (5) with complete documentation of the assessment methods used in evaluating DASH compliance; (6) with description on the degree of DASH compliance. All potentially relevant studies identified from the literature search were screened by the two independent investigators on the basis of study title and abstract. Duplicate studies and studies not meeting the above inclusion criteria were excluded. Full-text of the remaining studies was retrieved for further examination. Any disagreements on study selection were resolved by discussion or if necessary by the third investigator. To assess the methodological quality of the included studies, a valid international Quality Index was adopted [12]. The inter-rater reliability measured by the kappa statistics was 0.746, which considered as substantial agreement between the two investigators.

### Data extraction

Data of the characteristics of the study subjects, study design and duration, DASH assessment methods, and the degree of DASH compliance were retrieved independently by the two investigators in a standardized manner. Disagreements on data extract were discussed and referred to the third investigator when necessary. The primary measures of interest were the degree of DASH compliance and the corresponding assessment used.

## Results

**Figure 1:**
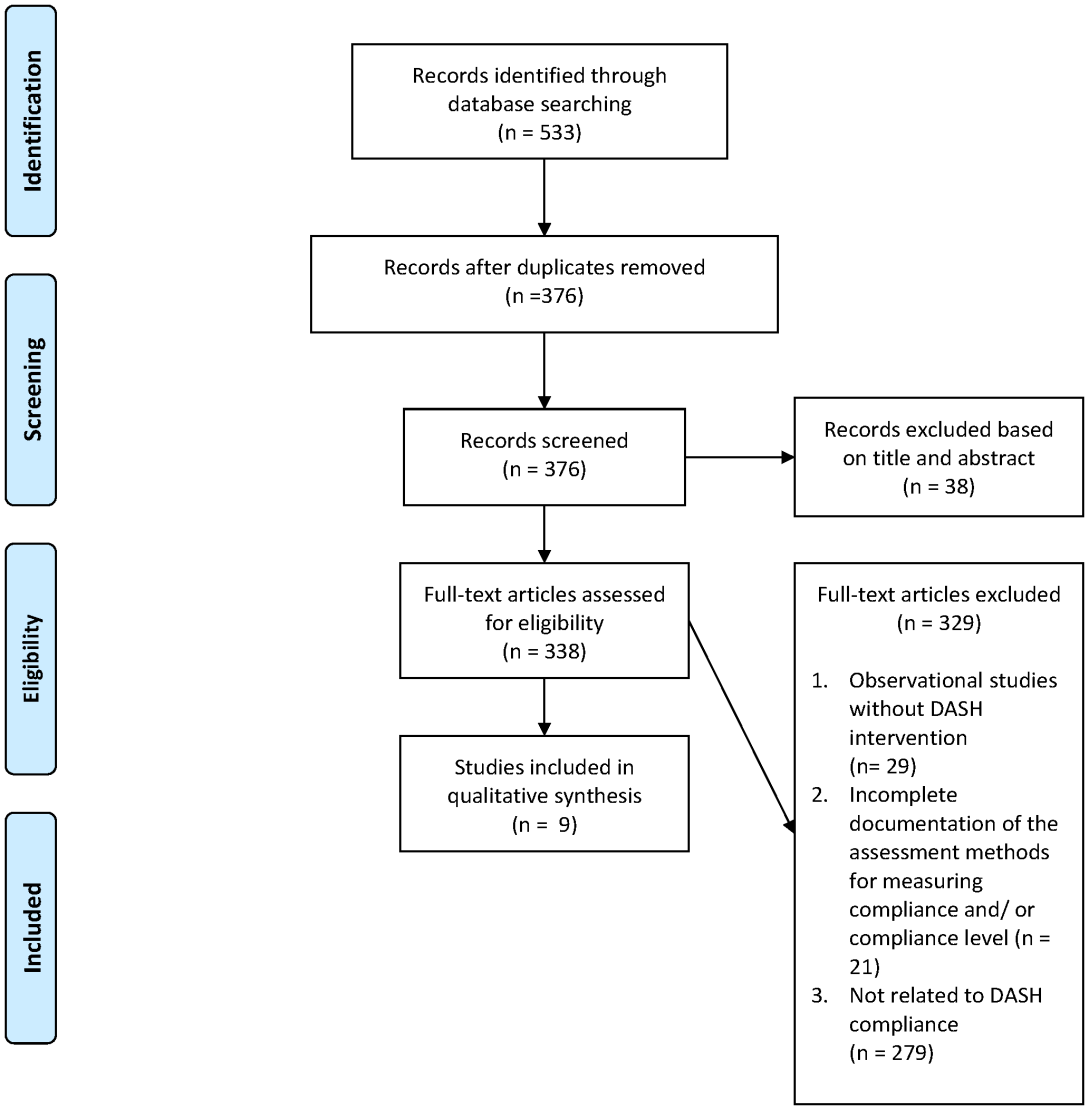
Flow diagram of study selection.

Limiting to studies published in English, a total of 533 studies were initially identified in these databases, in which 156 in MEDLINE, 220 in EMBASE, 88 in EBM Reviews (Cochrane Central Register of Controlled Trials, Cochrane Database of Systematic Reviews, and Cochrane methodology Register) and 69 in CINAHL Plus. After excluding duplicate studies on the basis of title or abstract, three hundred and thirty-eight publications were retrieved for more detailed review. Observational studies without DASH intervention, studies without complete documentation of the degree of compliance and the assessment methods used, and studies not related to DASH compliance were excluded. A total of 9 studies were included in this review (**Figure 1**). The assessment methods used and the DASH compliance level were used as the primary summary measures. The characteristics of these included studies were shown in **Table 1**.

**Table 1:** Characteristics of included DASH randomized controlled trials.

Author (Year)	Type of RCT	Duration (wk)	Sample size^a^	Mean age^b^ (SD)	Sex, Female^b^ (%)	Type of intervention	BP outcome, SBP/DBP (mmHg)	DASH compliance assessment method	DASH compliance level^c^
Booth et al. ^18^ (2003)	Parallel	12	54	47.7(9.5)	0	Counseling:	ΔBP from baseline*:	% of subjects meeting specific food group targets	
						1. DASH+Wt loss	1. -7.6/-5.4	Fruit	67%
						2. Low fat diet	2. -2.1/-1.0	Vegetables	48%
								Dairy	89%
Couch et al. ^9^ (2008)	Parallel	12	57	1: 14.3(2.1)	1: 38	Counseling:		% of subjects attained the pre-defined DASH dietary pattern	21%
				2: 14.4(2.1)	2: 36	1. DASH	1. 131.3/79.5 (Baseline); 120.9**/72.7 (Post)		
						2. Routine Care (RC)	2. 125/82.3 (Baseline); 123.1**/75.9 (Post)		
Epstein et al. ^14^ (2012)	Parallel	16	144	51.3(9)	67	Counseling:	ΔBP from baseline***:	Composite DASH adherence score	6.24 (maximum score of 10)^d^
						1. DASH+Wt loss	1. -16.1/-9.9		
						2. DASH	2. -11.2/-7.5		
						3. Usual diet	3. -3.4/-3.8		
Nowson et al. ^19^ (2009)	Parallel	14	95	1: 60.0(0.7) 2: 58.4(0.7)	1: 100	Counseling:	ΔBP from baseline:	24-hour urinary excretion	
					2: 100	1. Low sodium DASH	1. -5.6/-4.1	Sodium	Decreased (38.6±6.9 mmol/d)
						2. Low fat diet	2. -2.7/-2.9	Potassium	Increased (6.9±3.6 mmol/d)
Nowson et al. ^20^ (2004)	Cross-over	11	94	55.6(9.9)	40.4	Counseling:		24-hour urinary excretion	
						1. CD^e^	ΔLNAHK- ΔOD****:	Sodium	Decreased (20%)
						2. OD	-3.5/-1.9	Potassium	Increased (50%)
						3. LNAHK	ΔHC- ΔOD****:	Urea	Increased (23.0±9 mmol/d)
						4. HC	+3.1/+0.8		
Obarzanek et al. ^15^ (2007)	Parallel	72	782	50.0	62.0	Counseling:	ΔBP from baseline:	24-hour urinary excretion	
						1. Established^f^	1. -8.6/-6.0	Sodium	Decreased (24.5 mmol/d)
						2. Established +DASH	2. -9.5/-6.2	Potassium	Increased (9.6 mmol/d)
						3. Advice control	3. -7.4/-5.2	Phosphorus	Increased (10.7 mmol/d)
								DASH adherence index	Increase of 0.9 in score (0-1 indicates adherence to the DASH dietary pattern)
Racine et al. ^17^ (2011)	Parallel	52	147	72.5(8.3)	83.0	Counseling:		DASH score	1.85 (maximum score of 9)
						1. MNT^g^	NA^h^		
						2. Information			
Troyer et al. ^16^ (2010)	Parallel	52	210	≥60.0^i^	82.9	Counseling and meals provision:		DASH score	2.1 (maximum score of 9)
						1. Received meal^j^	NA^h^		
						2. Not received meal			
Windhauser et al. ^13^ (1999)	Parallel	11	459	45.0	47.0	Feeding trial:		Meal attendance	96.1%
						1. Control diet		Body weight	Maintained
						2. Increased fruit & vegetables (F/V)	ΔF/V- ΔControl*****: -2.8/-1.1	Urinary excretion	
						3. Combination/DASH	ΔDASH- ΔControl*****:	Sodium	Remained unchanged
							-5.5/-3.0	Potassium	Increased
								Phosphorus	Increased
								Urea Nitrogen	Increased
								Daily adherence score	Close to zero (zero indicated no deviation from the study diet)
								An anonymous post study survey	96.5% of subjects reported always or usually ate all of the study foods.

a Number of subjects included in the analysis.

b Data were reported based on treatment grouping if information of the full sample was not available.

c Compliance level in the DASH intervention group.

d Compliance level in the DASH diet alone intervention group.

e CD =  low potassium, low calcium diet; OD =  DASH diet; LNAHK =  Low sodium, high potassium diet; HC =  High calcium diet.

f Established behavioral intervention that promoted long-standing recommendations for BP control.

g Medical nutrition therapy (MNT) on DASH diet knowledge.

h No BP measure was described in the included studies of this review and related studies in the literature.

i No mean age was reported. Only mentioned the inclusion criteria as age ≥60 years old.

j Meals were developed by using the American Dietetic Association MNT protocols for caloric and nutrient content requirements for the specific diagnoses, guidelines outlines in the DASH diet, and Administration on Aging Nutrition Program dietary requirements.

*Between group differences in ΔBP (*p* = 0.001).

**Between group differences in SBP reduction (*p*<0.01).

***Between group differences in ΔBP (*p*<0.001).

****Between LNAHK and OD diet period differences in ΔSBP (*p*<0.001), ΔDBP (*p*<0.05). Between HC and OD diet period differences in ΔSBP (*p*<0.01).

*****Between group differences in mean change (ΔDASH- ΔControl) in SBP and DBP (*p*<0.001). Between group differences (ΔF/V- ΔControl) in mean change in SBP (*p*<0.001).

### Feeding trials

In the original DASH feeding trial of 459 free-living persons, excellent adherence was reported by both objective and subjective measures [13]. The DASH trial was conducted to assess the effects of three dietary patterns on BP, including control, increased fruits and vegetables, and combination diets. Subjects attended 3 weeks run-in of control diet, followed by 8 weeks of intervention feeding. Non-study foods and beverages were restricted. Objective adherence measures included meal attendance, measurement of body weight, and urinary excretion (sodium, potassium, phosphorus and urea nitrogen). Daily diaries and an anonymous post-study survey were used to assess subjective dietary adherence. An overall adherence score was also formulated, ranging from 0 to 2. Based on daily diary, participants consumed all the study foods on 96.0% and 97.9% of the study days during the run-in and feeding period, respectively. Similar levels of adherence were obtained from anonymous surveys. The overall mean adherence score also indicated a nearly perfect adherence.

### Other intervention studies

Various methods were used in the literature to assess the DASH compliance in the intervention studies, including construction of DASH score based on dietary intake, assessment of achievement of defined DASH dietary patterns, and the examination of urinary excretion.

#### a. Compliance level measured by DASH score

A scoring system was adopted by several studies to assess the DASH compliance. The recent ENCORE study used a composite index of adherence, the DASH Adherence Score, for assessment [14]. This was a 4-month clinical trial enrolled with 144 overweight and obese adults with suboptimal BP (SBP: 130–159 mmHg, and/or DBP: 85–99 mmHg). Participants were randomized into 1 of the 3 groups: DASH alone (DASH-A), DASH plus behavioral weight management (DASH-WM), or usual diet control (UC). Weekly 30–45 minute group sessions introducing the DASH eating plan, and discussing goal setting and action plans were provided to both the intervention groups. Additional calorie restriction, behavior modification, and aerobic exercise sessions were included in the DASH-WM group. The UC group was asked to keep their daily routine for diet and exercise. The DASH adherence score was constructed based on dietary information from a validated food frequency questionnaire (FFQ). This was a composite score, composing of sub-scores from 10 food and nutrient components (grains; fruits; vegetables; nuts, seeds, and legumes; dairy; meat; fat; saturated fat; sweets; and sodium). A score of 0 to 1 was given to each component based on intakes compared to the recommendations, yielding a maximum total score of 10. The mean post treatment total adherence scores were 6.60, 6.24, and 3.62 in the DASH-WM, DASH-A, and UC group respectively.

Another DASH Adherence Index was constructed by Obarzanek et al. to measure the overall compliance to the DASH diet in the PREMIER trial [15]. PREMIER was a randomized controlled trial with 18 months intervention period. Seven hundred and eighty two participants aged ≥25 with prehypertension or stage 1 hypertension were randomized into 1 of the 3 treatment groups: “Established” (receiving established recommendations for BP control), “Established Plus DASH” (receiving established recommendations and DASH diet recommendations), or advice control group (receiving information about lifestyle factors that control BP). Participants in the “Established Plus DASH” group were encouraged to achieve the DASH goals, which included increasing daily intake of fruits and vegetables (9–12 servings), and low-fat dairy products (2–3 servings), while reducing intake of saturated fat (≤7% energy). Three sub-indices were constructed based on two 24-hour dietary recalls in relation to the above 3 goals. A score of 0–1 was assigned when intake was within the target range. Participants with intake better than the target range were given a score higher than 1, while those with worse intake were given with a score below 0. The average of these 3 sub-indices represented the overall DASH adherence. At 6 months and 18 months, a near zero adherence score was reported in the DASH group, which was higher than that in the other 2 treatment groups.

Under another randomized prospective trial, 2 studies examining the effects of different interventions on DASH compliance using DASH score were identified [16], [17]. This trial involved 210 participants aged ≥60 years with hyperlipidemia and/or hypertension. Participants were randomized into 4 groups for 52 weeks, including information group, therapeutic meals group, medical nutrition therapy (MNT) group, and MNT plus therapeutic meals group. Dietary intakes were assessed using 24 hour recalls. The DASH score was calculated based on the recommendations for 9 nutrients (protein, total fat, saturated fat, cholesterol, fiber, magnesium, calcium, potassium, and sodium). Meeting a nutrient target scored one point, and a maximum total score of 9 could be obtained. An intermediate DASH score was also calculated, in which meeting an intermediate target and a nutrient target gained 0.5 points and 1 point, respectively.

The study by Troyer et al. [16] was the main study focusing on the dietary changes between those received and not received therapeutic meals, while the study by E. Racine et al. [17] was the sub-analysis of the effectiveness of MNT versus education information on DASH diet knowledge. In the first study [16], the analysis involved 210 participants, in which 50% received 7 therapeutic meals per week for 52 weeks. Participants receiving meals obtained a DASH score of 1.4±0.9, 2.3±1.5 and 2.1±1.6 at 0 month, 6 months, and 12 months respectively. They were 20% more likely to have intermediate DASH accordant at 6 months than those who did not receive meals (*p* = 0.001).

Racine et al. [17], on the other hand, analyzed 147 participants received either information or MNT. Information group received mailed brochures/educational factsheets regarding disease management. Three sessions of MNT were provided to the MNT group by registered dietitians, consisting of nutritional assessment, identifying treatment goals, giving nutrition prescription, and offering self-management training. At baseline, the mean DASH scores for the information group and MNT group were 2.03±1.53 and 2.36±1.63 respectively. Participants in the information group obtained a DASH score of 2.20±1.80 at 6 months and 2.40±1.95 at 12 months; while those in MNT group got a DASH score of 2.16±1.53 at 6 months and 1.85±1.43 at 12 months.

#### b. Compliance level measured by achievement of the defined DASH dietary pattern

A 3-month randomized intervention study of 57 adolescents with either hypertension (SBP and/or DBP >95th and <99th percentile for age, gender and height) or prehypertension (SBP and DBP between 90th and 95th percentile for age, gender and height) documented 21% of the subjects attained DASH dietary pattern after intervention [9]. The intervention consisted of an initial 60 minute face-to-face dietary counseling with a dietitian about the DASH eating plan, giving specific number of serving recommendations for the major food groups. Eight weekly and 2 biweekly phone calls, and biweekly mailings were provided to the participants. They also received incentives when meeting the DASH dietary goals. On the other hand, subjects in the control group were given routine nutrition care with a single 60 minutes counseling session about general recommendations for hypertensive patients, which included reduction of dietary sodium and controlling body weight. No specific serving recommendations were provided. Dietary compliance was assessed based on the defined DASH dietary pattern (≥8 servings of fruits and vegetables/day; 3 servings of low fat dairy foods/day; <30% of calories from fat).

Compliance to the DASH diet was evaluated based on individual intake target for 3 food groups (fruits, vegetables and dairy products) in the randomized trial by Booth et al. [18]. This was a 12 weeks randomized trial involving 54 male participants to compare the effects of a DASH type diet and a low fat (LF) diet on reducing the cardiovascular risk factors in the context of weight loss. All participants received 5 face-to-face visits and 2 phone calls during the study. Subjects in the DASH group were taught to follow specific servings of food groups emphasized in the DASH diet (≥4 serves of vegetables, ≥4 serves of fruits, ≥3 serves of dairy, ≤4 serves of fats per day. 4 serves of unsalted nuts, ≥3 serves of fish, 1 serve of legumes, ≤2 serves of red meat per week). General dietary advices to limit high fat foods and to increase fruits and vegetables were given to the LF group, with no specific daily intake target emphasized. FFQ was adopted to assess the food group intake. In the DASH group, the percentages of subjects meeting the target in fruits, vegetables, and dairy group at week 12 were 67%, 48% and 89% respectively. A lower proportion of LF participants achieved the intake target in these 3 food groups (fruits: 48%, vegetables: 30%, dairy: 37%).

#### c. Compliance level measured by urinary excretion

Two randomized trials used urinary excretion to measure the dietary compliance to DASH. A study by Nowson et al. [19] reported a high level of compliance as evidenced by the significant reduction in urinary sodium (38.6±6.9 mmol/d) and increase in potassium (6.9±3.6 mmol/d). In this study, 95 postmenopausal women (SBP: ≥120 and <160 mmHg, DBP: ≥80 and <95 mmHg) were randomized to receive dietary instructions for either low sodium DASH diet with a low dietary acid load including lean red meat on most of the days, or a control diet with high acid load for 14 weeks. Participants received dietary counseling by registered dietitian for 5 times and had 2 telephone contacts during the study period. The intervention group was advised to follow specific recommendations for the key DASH food groups. Lean red meat, and several low-sodium or salt-free foods were provided to the participants. The control group, on the other hand, received advice on healthy diet, and increasing intake of cereals and breads with high acid load. Regular-salt foods were given to this group.

The other randomized trial with 94 hypertensive subjects (SBP: ≥120 mmHg; DBP: ≥80 mmHg) documented a 20% reduction in urinary sodium and around 50% increase in urinary potassium during the DASH diet period [20]. This study examined the effects of three different diets including DASH, low-sodium and high potassium, and high calcium diet on BP. There was 1-week run-in period at baseline. Subjects received two 4-week intervention periods of the assigned diet with 2-week control periods in advanced. Initial dietary counseling was provided for 10–30 minutes. Subjects were contacted every 2 weeks to reinforce the dietary changes. Intake recommendations for different food groups were given based on the diet that they were assigned to. For example, those in the DASH diet group were instructed to have ≥3 servings of low-fat dairy, ≥8 servings of fruit and vegetables daily, and also include ≥3 servings of fish, ≥1 serving of legumes, and ≥4 servings of unsalted nuts and seed weekly. They were asked to limit red meat to ≤3 servings per week.

## Discussion

This review demonstrated there were a great variety of methods used in assessing DASH compliance, and there was no existing gold standard consensus for the best approach. Both objective and subjective methods were identified. Each method may have advantages, as well as disadvantages. Urinary excretion is the most objective biochemical indicator for the dietary intake of several nutrients. It is easily accessible and is an assessment method that is independent of the respondent's memory [21]. However, there are factors affecting the agreement between the urinary excretion and dietary intake, including the completeness of sampling and the hydration status of subjects [22]. Also, the assessment of urinary excretion might only reflect intake of several nutrients, failing to capture the overall dietary consumption. In contrast, using DASH score or achievement of the defined DASH dietary pattern is a subjective approach to assess DASH compliance. This method uses self-reported data of dietary intake obtained by dietary recalls or FFQ to compare with recommendations. This enables the researchers to attain additional information on the type and quantity of foods consumed by individuals, indicating the overall diet quality. However, this is subject to the inherent limitations of the dietary assessment methods used, for example, the response bias and recall bias [21]. It will be the most comprehensive if both objective and subjective methods were used. The objective measurement serves as a validity check for the subjective dietary assessment. However, the choice of the most appropriate assessment method depends on the study outcomes, the characteristics of the study population, and the resources available.

Although it was less possible to directly compare the compliance level among the included studies due to the heterogeneity in the methodology, it was found that the degree of compliance varied with the study design. It is of no surprise that excellent adherence was reported in the DASH feeding trial as food was provided to the patients. However, when it changed to a “real world setting” where participants were given dietary advice for DASH instead of providing food items, a lower level of adherence was reported. The ENCORE study reported a mean DASH score of around 6 (out of a maximum 10) in both the intervention groups with DASH [14], while studies by Troyer et al.[16] and Racine et al.[17] reported a score of approximately 2 (maximum: 9) after intervention. Only 21% of the participants achieved the DASH targets for fruits, vegetables, and saturated fat after dietary counseling in a 3-month randomized intervention study [9]. Moreover, decrease in compliance with time after intervention was noticed in 4 of the studies reviewed [9], [15]–[17], indicating it was also challenging to maintain the adherence to specific dietary advice. However, the standard for good DASH compliance was not well defined and the judgment for the level of compliance remained uncertain.

Despite the fact that patients might not have perfect adherence to the DASH diet, previous studies showed significant improvements in dietary intakes among subjects with dietary intervention. Participants in the PREMIER study showed a significant decrease in total fat, saturated fat and cholesterol intake, while increase in intake of dietary fiber and many vitamins and minerals after intervention [23]. Similar improvements in dietary fat and minerals such as potassium, magnesium and sodium were demonstrated in other studies [9], [20]. Dietary improvements were accompanied by positive BP changes [9], [18]–[20], [24]. The recent ENCORE study demonstrated that DASH adherence score was an independent predictor of SBP, in which every 2-point increase in DASH adherence score was associated with a reduction of 3.4 mmHg in SBP [14]. This is of great importance in the management of hypertension. In the recent 7^th^ report of the Joint National Committee on Prevention, Detection, Evaluation, and Treatment of High Blood Pressure, it was estimated that a 5 mm Hg decrease in SBP in the population would result in overall reduction in mortality due to stroke, coronary heart disease and all-cause mortality by 14%, 9% and 7% respectively [3]. Other prospective cohort studies in the literature revealed that a diet more consistent to DASH was associated with a lower rate of heart failure [25], [26], a lower risk of coronary heart disease and stroke [26], and a lower incidence of diabetes [27]. All these provide supporting evidence to promote the adoption and adherence to this dietary plan.

The major limitations of the present review were the scarcity of eligible studies and the substantial variability between them. Only very few studies were found in the literature consisting of both the assessment method for DASH compliance and an intervention arm for DASH. Many studies were observational in nature, examining the association between the consistency with the DASH diet and risk of developing different diseases. The very few studies identified also differed in study design and outcomes. This inherent lack of homogeneity between the included studies made it hard to perform a meaningful comparison. In addition, the restriction of search results to English language might lead to the exclusion of potentially relevant studies and this contributed to another limitation of the current review.

## Conclusion

This systematic review outlines a wide range of methods used to assess the DASH compliance level, which is an indispensable part in evaluating the impact of the dietary intervention. However, no consensus existed regarding the best approach. Despite the heterogeneity in the methodology and the study design, compliance level to the DASH diet was generally low when only counseling service was given without food supplies. Given the potential health benefits of this dietary plan, there is a need for future research to establish the gold standard for examining DASH compliance, and to study the barriers for adopting dietary changes. It might also be of interest to develop and integrate an individual compliance assessment component into the education program, serving as a self-monitoring tool to enforce the implementation of dietary modifications. Effective strategies are warranted to enhance and maintain the adherence to the recommendations, maximizing its potential benefits on BP control.

## Supporting Information

Table S1 Search strategy in MEDLINE database.
(DOC)
