# Supplementary material for: Compliance with the Dietary Approaches to Stop Hypertension (DASH) Diet: A Systematic Review
Source: PLoS One. 2013 Oct 30;8(10):e78412. doi: 10.1371/journal.pone.0078412 (PMC3813594; doi:10.1371/journal.pone.0078412)
Supplement: Table S1 — Search strategy in MEDLINE database. (DOC) [file pone.0078412.s001.doc]

Table S1- Search strategy in MEDLINE database

Database(s): Ovid MEDLINE(R) 1946 to December Week 4 2012
Search Strategy:

| **#** | **Searches** | **Results** |
| --- | --- | --- |
| 1 | (DASH or dietary approaches to stop hypertension).af. | 4602 |
| 2 | (compliance or adherence or consistency or concordance).af. | 247064 |
| 3 | 1 and 2 | 206 |
| 4 | limit 3 to yr="1992 - 2012" | 203 |
| 5 | limit 4 to english language | 193 |
| 6 | remove duplicates from 5 | 156 |
